# Disentangling Coordination among Functional Traits Using an Individual-Centred Model: Impact on Plant Performance at Intra- and Inter-Specific Levels

**DOI:** 10.1371/journal.pone.0077372

**Published:** 2013-10-09

**Authors:** Vincent Maire, Nicolas Gross, David Hill, Raphaël Martin, Christian Wirth, Ian J. Wright, Jean-François Soussana

**Affiliations:** 1 INRA Grassland Ecosystem Research (UR 874), Clermont-Ferrand, France; 2 INRA, USC Agripop (CEBC-CNRS), F-79360, Villier-en-Bois, France; 3 CEBC-CNRS (UPR 1934), F-79360, Villier-en-Bois, France; 4 CNRS LIMOS (UMR 6158), Blaise Pascal University, Aubière, France; 5 Universität Leipzig, Institut für Biologie I, Leipzig, Germany; 6 Department of Biological Sciences, Macquarie University, New South Wales, Australia; University of Leipzig, Germany

## Abstract

**Background:**

Plant functional traits co-vary along strategy spectra, thereby defining trade-offs for resource acquisition and utilization amongst other processes. A main objective of plant ecology is to quantify the correlations among traits and ask why some of them are sufficiently closely coordinated to form a single axis of functional specialization. However, due to trait co-variations in nature, it is difficult to propose a mechanistic and causal explanation for the origin of trade-offs among traits observed at both intra- and inter-specific level.

**Methodology/Principal Findings:**

Using the Gemini individual-centered model which coordinates physiological and morphological processes, we investigated with 12 grass species the consequences of deliberately decoupling variation of leaf traits (specific leaf area, leaf lifespan) and plant stature (height and tiller number) on plant growth and phenotypic variability. For all species under both high and low N supplies, simulated trait values maximizing plant growth in monocultures matched observed trait values. Moreover, at the intraspecific level, plastic trait responses to N addition predicted by the model were in close agreement with observed trait responses. In a 4*D* trait space, our modeling approach highlighted that the unique trait combination maximizing plant growth under a given environmental condition was determined by a coordination of leaf, root and whole plant processes that tended to co-limit the acquisition and use of carbon and of nitrogen.

**Conclusion/Significance:**

Our study provides a mechanistic explanation for the origin of trade-offs between plant functional traits and further predicts plasticity in plant traits in response to environmental changes. In a multidimensional trait space, regions occupied by current plant species can therefore be viewed as adaptive corridors where trait combinations minimize allometric and physiological constraints from the organ to the whole plant levels. The regions outside this corridor are empty because of inferior plant performance.

## Introduction

Functional traits are any morphological or physiological attributes with significant effects on plant fitness [1]. Many functional traits do not vary independently but rather form groups of co-varying traits, sometimes known as strategy spectra (or dimensions / axes of ecological / evolutionary specialization in Diaz et al. [2]). One main objective of functional ecology is to quantify these correlations to investigate the mechanisms (e.g. trade-off) underlying the coordination of traits within and between species, and to relate these trait dimensions back to dimensions of plant ecological strategy [3].

One trait-strategy spectrum has become known as the *leaf economic spectrum* [4]. This spectrum runs from species with cheaply constructed leaves with high nutrient concentrations and fast physiological rates but short leaf lifespan (often dominating soil N rich environments), to those with sturdier longer-lived leaves, with slower physiological rates and lower nutrient concentrations (often dominating poor environments [5,6]). Other key trait-strategy spectra include those associated with plant stature, which imply allometric constraint between branching or stem / tiller density (e.g. number of tillers per length of stem) and leaf / plant size [6,7], and the manner in which reproductive resources are divided into many-small versus few-large propagules [8]. In theory, each strategy spectrum represents an independent dimension by which plant species can differentiate into separate niches [9], with important implications for species coexistence, community assembly and ecosystem functioning [10-12]. How these independent strategy spectra interact at the intraspecific level to determine plant performance may be of primary importance to understand the coordination of traits, as revealed by Vasseur et al. [13] on a single species.

Both phenotypic plasticity and natural selection are likely to explain within-species trait variability observed in the field [14]. (Here, we broadly define phenotypic plasticity as the capacity of a given organism to alter its morphology and / or physiology in response to environment; and selection as referring to selection of particular genotypes with particular trait values at the population level.) Trait plasticity has been proposed as a key parameter for plant fitness [15,16]. It can promote plant persistence in response to the environment changes [17] and it is an important mechanism for community assembly [18,19]. Intraspecific trait variation has often been shown to be idiosyncratic, i.e. trait- and species- dependent [20,21] and has sometimes been hypothesized to be part of a species ecological strategy [22]. To date, few studies have specifically tested this proposition (but see 23). In addition, it is unclear whether intraspecific variation obeys the same allometric or physiological trait coordination as the interspecific variation along strategy spectra [24,25] and, finally, how trait coordination would affect species ability to be plastic.

Because traits covary it is difficult to isolate the role of individual traits on ecological processes. For instance, by a model approach, Osone et al. [26] have shown that the correlation between relative growth rate and specific leaf area requires associations of specific leaf area with nitrogen absorption rate of roots. Two broad types of modeling approaches have been proposed to achieve such understanding: i) statistical approaches, investigating at lower levels of biological organization the causality in the relationships among traits [27,28]; and ii) simulation approaches, which involve breaking the correlation between traits observed in nature and then quantifying impacts on a given process [29,30]. These sorts of approaches (e.g. [31]) may help to quantify the isolated effect of a particular trait at the organ to the whole-plant level, and understand whether a suite of correlated traits improve, say, resource acquisition and utilization compared to the effect of a single varying trait.

Current simulation approaches that used to investigate the causal mechanisms underlying trait co-variations, rarely takes into account the role of plant morphogenesis, i.e. ontogenetic change in morphology and stature (but see 32 for only one species). Yet, interactions between structural architecture and resource allocation to root versus shoot could be key to investigating the coordination between traits at the intraspecific level and how they emerge at the interspecific level. Here, we use a mechanistic model (Gemini [33]) to do that. Gemini uses plant functional traits as parameters, explicitly connects the plant morphogenesis with the capture, allocation and utilization of carbon and nitrogen, and has been calibrated and evaluated on 12 perennial grass species [16], which are common within semi-natural mesic grasslands in Europe [34].

We explored the influence of two particular sets of traits: two leaf traits (SLA and LLS), which are correlated negatively along the leaf economics spectrum [4]; and two plant stature traits, which vary independently from the leaf economic spectrum among grass species (plant height, H and tiller density, TD) and have been shown to be negatively correlated due to allometric rules (avoidance of self-shading [6]). Based on the hypothesis that co-variations among traits are relevant for plant performance (i.e. are not random in the sense of Turnbull et al. [35]), we test at the intraspecific level two hypotheses:

1 In a given environment, exists for each species an optimal trait combination that maximizes plant performance. Since plant processes are coordinated for this optimal trait combination, plant performance declines dramatically when trait values move away from this optimum.
2 In response to an environmental change, changes in trait values may be needed to restore the coordination of plant processes. Such variations can be predicted from the principle of plant performance maximization.

Emerging from intraspecific level, we predict that at the interspecific level:

3 Strategy spectra are independent, e.g leaf economics (SLA vs. LLS) is independent from Corner’s rule (H vs. TD).
4 Species positions along strategy spectra affect both the maximal plant performances and the trait plasticity.

To test these hypotheses with the Gemini model, we ran a simulation experiment within the 4*D* trait space defined by the two leaf and two plant stature traits. While systematically exploring this trait space we broke correlations observed in nature across these traits. We simulated plant performance responses to trait variation and demonstrate the occurrence of a species-specific single trait combinations maximizing plant performance. We compared these predicted optimal trait combinations to trait values measured under field conditions.

## Methods

### Grassland Ecosystem Model with Individual ceNtered Interactions, Gemini

Gemini was fully described by Soussana et al. [33,36]. It is used to understand how biotic and abiotic factors affect plant population dynamics and the C-N cycles of one and many interacting populations in grasslands. The abiotic factors modeled are climate and common management-related conditions in grasslands (cutting, grazing and fertilization). Biotic factors include the diversity of herbaceous plants communities. The model tracks the acquisition and the utilization of resources (photosynthetically active radiation and inorganic nitrogen) for plant growth and survival. Recruitment from seeds, immigration of new populations, and survival in response to severe environmental stress, are not considered by the model.

Gemini consists of vegetation and soil sub-models, coupled with environment and management sub-models. The vegetation sub-model, Canopt is an individual-centered model of pasture species growth that simulates the dynamics of a plant population made up of average individuals. Population turnover, shoot and root morphogenesis, photosynthesis, respiration, transpiration, N acquisition by uptake, allocation of assimilates between structural compartments, and reserve storage and remobilization, are simulated for each plant population within multi-species canopy layers. Four modules are assembled. First, a *plant physiology and partitioning module* simulates the acquisition and the balance of C and N substrates. Partitioning of growth between shoot structures, leaf photosynthetic proteins and roots is based on two assumptions: (i) functional balance between root and shoot activity [37], (ii) coordination of leaf photosynthesis [38,39]. The corresponding state variables are the biomass of the three structural compartments, of one substrate C-N pool and of two reserves C and N pools. Corresponding parameters define the chemical composition of plant tissues and physiological rates of resources acquisition and utilization. According to a supply/demand law for the utilization of C and N substrates, the physiological module is coordinated with the second module, a *morphogenesis module*, which computes the demography, the shape and the size of leaves and roots, as well as plant axes demography (e.g. tillers for grasses) [40,41]. Tillers are interconnected within a plant and share C-N substrates that affect the dynamics of the population. The corresponding state variables are the length and number per plant axis of leaves and roots, and the number of plant axes per unit ground area. A third *environment module* computes the radiative and N balances among soil and canopy layers. Finally, a *management module* runs discrete events creating disturbance (by cutting and/or grazing) and supplying nutrients (N fertilizer supply).

Gemini allows one to investigate the details of physiological and morphological processes that drive species responses to trait variations (see Figure S1), such as: light interception; net photosynthesis; inorganic N uptake capacity; specific root area; partitioning coefficients of C and N substrates between shoot structures and roots (*P*) and between shoot structures and leaf proteins (*Q*); and the C:N ratio of labile substrate pools. In Gemini, the C and N substrate pools correspond to labile carbohydrates and to NO_3_^-^, NH_4_^+^ and reduced soluble N, respectively, and their mass balance (*W*_C,_ *W*_N_) results from the dynamics of the following plant processes (see 33 for details):

*W*_C_/dt = Photosynthesis + Remobilisation -Respiration - Partitioning -Storage - Exudation

*W*_N_/dt = Uptake + Fixation + Remobilisation -Partitioning - Storage - Exudation

As such, the total amount of substrates (*W*_C_ + *W*_N_) and the C:N ratio of plant substrates are *in-planta* markers of coordination between ecophysiological processes determining plant performance. For a given species under a given environment, these markers should fluctuate within rather narrow boundaries in order to maximize plant performance [33].

Plant functional traits measured under close to optimal environmental conditions are required to calibrate the Gemini model [33]. As such, the values used to calibrate SLA, LLS and H traits correspond to potentials that species are likely to reach under favorable conditions in the field. As tiller density (TD) is a state variable of the model, its calibration is different and corresponds to the mean value of TD observed in the field two or three years after establishing a grass monoculture. During the simulation, SLA, LLS and H may each vary in response to environmental conditions. Such variations are constrained by the corresponding potential trait values that vary according to the genetic background of the plant population. In contrast, TD variations are not constrained by a potential TD value. A detailed list of all 132 equations, as well as the variables and the default parameter values is available at www1.clermont.inra.fr/urep/modeles/gemini.htm. The four studied traits refer specifically to the morphogenesis module. They all have an indirect impact on C and N internal fluxes within the plant through the coordination between the physiology and the morphogenesis Gemini modules. A brief review of their implication in model equations is given in Text S1.

### Field measurements and model parameterization

Eleven C_3_ grass species and one cultivar were studied in field monocultures from 2003 to 2004 (see 42 for details): *Alopecurus pratensis*, *Anthoxanthum odoratum*, *Arrhenatherum elatius*, *Dactylis glomerata*, *Elytrigia repens*, *Festuca rubra*, *Holcus lanatus*, *Lolium perenne*, *Phleum pratense*, *Poa pratensis*, and *Trisetum flavescens* and the *Lolium perenne* cultivar, Clerpin. These species co-occur in productive grasslands but they differ in their abundance patterns in response to disturbance and soil fertility [12]. They are among the 20 most widely distributed *Poaceae* species in the French Massif Central region. Trait measurements were done in previous field studies for model parameterization and evaluation [33]. The complete experimental design comprised 72 monocultures arranged in a complete randomized block design with two levels of N fertilization (120 and 360 kgN ha^-1^ yr^-1^) (see also Text S1 for a detailed description of the experimental design, traits measurements and plant performance).

Overall, the Gemini model requires a total of 64 parameters, including 27 species specific parameters; of these, twelve are related to shoot morphology; seven to root morphology; four to chemical composition and four to physiology [33]. Values of all species specific parameters were derived from above and below-ground trait measurements on the eleven native grass species and on the *Lolium* cultivar grown in field monocultures under high N availability. Two parameters (fine root maximum length and fine root lifespan) were optimized by maximizing axis biomass (*W*_G_). This first optimization was done under high N management treatment keeping a constant axis density for each species. A second optimization was run for the two population demography parameters (apparition and senescence rate of axes) by fitting simulated with measured tiller density (TD) per unit ground area. Changing the value of these two parameters did not affect the outcome of the virtual experiment presented in this paper (data not shown).

### The virtual experiment

Gemini was used to test the effect of trait variation on plant performance both at the inter- and intra-specific levels. As the reproduction of the selected grass species is mainly vegetative, plant performance was estimated in the model via annual biomass production, which itself should be a good proxy of plant fitness [33]. The role of the two leaf traits (specific leaf area, SLA and leaf lifespan, LLS) and the two plant stature traits (plant height, H and tiller density, TD) was studied (see Text S1 and Table S1 for details). A sensitivity analysis was made by varying model parameters that were either identical to the traits, or represent simple mathematical functions of them. Variation in SLA was achieved by changing the leaf dry-matter content (LDMC) parameter. For each species, an allometric relationship was derived, considering a constant leaf thickness and constant ratio between sheath and leaf lengths (Text S1). Variations in plant height *H* were achieved by changing the potential length of mature leaves *L*_*0*_ (cm). For each species, plant height was calculated considering a dynamic leaf shape, according to the plant population density. Variations in LLS were achieved by changing the phyllochron (thermal time, in degree days, between the appearance of two successive leaves, Ph). These two variables were closely correlated in nature [43] and within the 12 grass species over the year (data not shown). Finally, the initial tiller number (*TD*_*0*_, tillers m^-2^) is simply the initial value of the state variable.

A fully crossed sensitivity analysis was conducted to explore the simulated dynamics of plant vegetative growth in monoculture in response to variation in each of the four traits, each trait being a factor in the experimental design (4*D* trait space). For each species the model parameters reflecting the four traits were varied in ten equidistant steps (Table S1). The step values for each species were determined between minimum and maximum boundaries, which were selected to obtain for each trait a ±30% variation around the species’ trait mean value observed in the field. In addition to the ten predefined steps, simulations with the measured values of each trait in the field monocultures were run. This design was applied at two N availability levels corresponding to the fertilization treatments in the field experiment. Climatic data (radiation, temperature, precipitation and air moisture), recorded during the field experiment in 2003-2004, were used to run the model. Management data recorded during the experiment (cutting dates and timing and amounts of N fertilizer supply) were used for model simulations. Each simulation ran over ten years (repeating the 2003-2004 climate data five times), a necessary running time to check for the stability of the model response. In addition, simulations started from a quasi-equilibrium state which was obtained by spin-up model runs. The two simulation campaigns (N+ and N-), corresponding to more than 350 000 simulations, took 30 days on a Symmetric multiprocessor with 8 AMD 64 bits dual core, 256 Gb. of RAM under the Centos 4 operating system. The Gemini software proved to be extremely reliable since: (i) more than 99.99% of all simulations were executed without error; (ii) plant growth showed high stability over the 10 simulated years (data not shown).

### Data analyses

**Figure 1:**
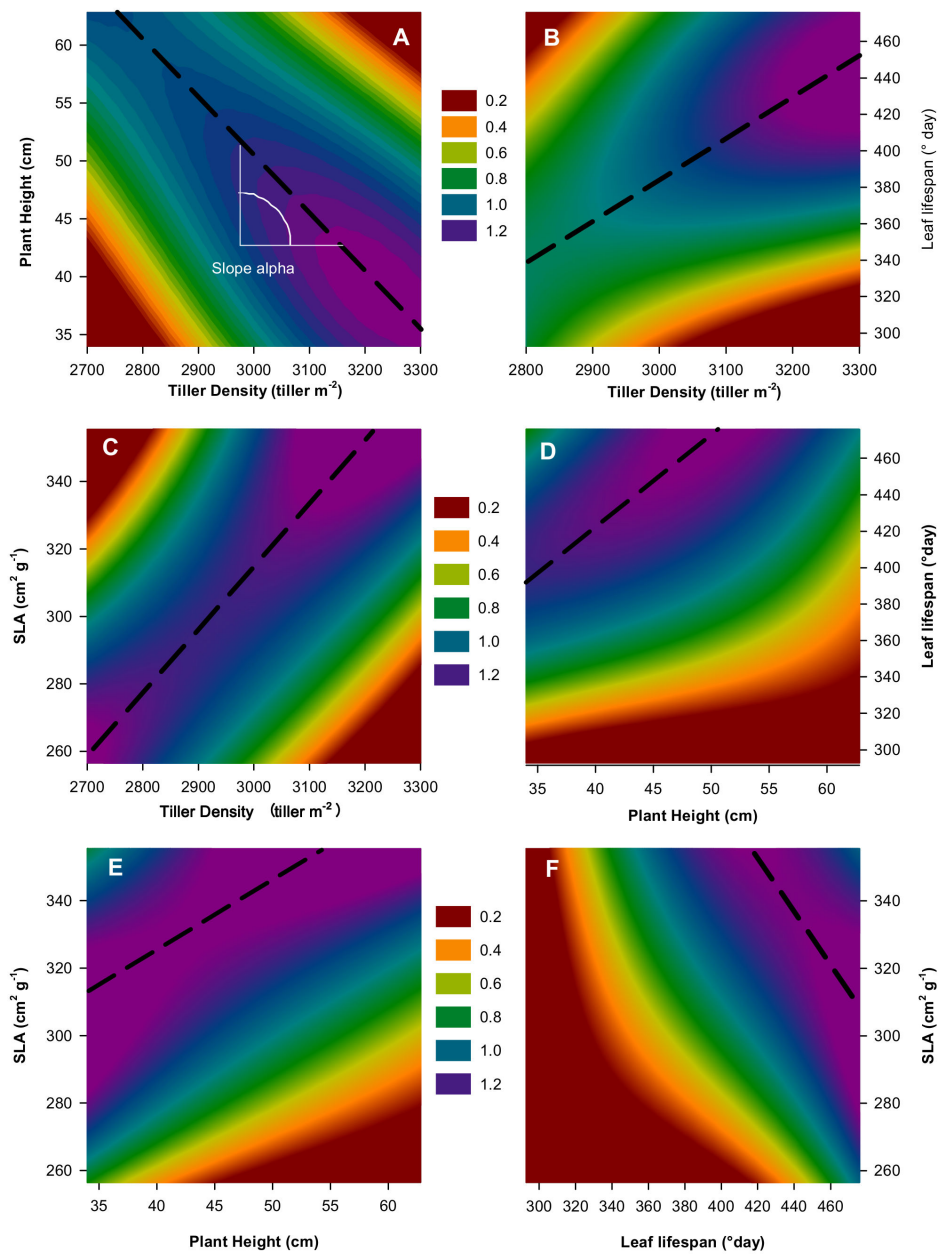
Simulated effects of trait variations on plant annual biomass production (g plant^-1^) for *Arrhenatherum elatius* in the high N treatment. (A) Tiller density *vs* Plant Height; (B) Tiller Density *vs* Leaf Lifespan; (C) Tiller density *vs* Specific Leaf Area; (D) Plant Height *vs* Leaf Lifespan; (E) Plant Height *vs* Specific Leaf Area; (F) Leaf Lifespan *vs* Specific Leaf Area. In each 2*D* plot, the values of the two remaining traits were fixed to the species’ mean trait value observed in the field. For each pair-wise trait combination, a dashed line indicates a ridge along which trait co-variation maximizes annual biomass production. The slope (α_i,j_) of the corresponding linear regression characterizes the relationship between the (*i*, *j*) trait pairs as predicted by the model at the intraspecific level.

In one simulation run, i.e. for each trait variation step, the annual biomass (below- and aboveground) was recorded for each simulated year and then averaged over the ten years simulation period for each species. For each species and for each N level, we generated an adaptive landscape in which the dynamic of species performance could be explored through the independent variation of the four traits. Within this landscape, we were able to record:

- In a 4*D*-trait space, the single combination of trait values (*trait_max_*) that maximized the vegetative growth (adaptive peak, Table 1),
- In the various 2*D*-trait spaces and under high-N conditions, the *slopes* α that each described a set of equally-optimal trait combinations (‘adaptive ridges’). These ridges can also be thought of describing the degree to which a trait can vary independently from the others with only a limited impact on plant performance (see dashed lines in Figure 1A as an example for *A. elatius* and Table 2 for the *slopes* α values of the six trait-pairs among the 12 species).

**Table 1:** *Trait*_*max*_ values predicted by the model in high N conditions and optimal C:N ratio of substrates within the plant species; SLA, Specific Leaf Area; H, Plant Height; LLS, Leaf Lifespan; TD, Tiller Density.

**Species**	**SLA**	**H**	**LLS**	**TD**	**C:N ratio**
	(*cm* ^*2*^ * g* ^*-1*^)	*(cm)*	*(°day)*	*(tiller m^-2^)*	*(gC g^-1^N)*
*A. pratense (Ap)*	263	56.8	549	2591	7.01
*A. odoratum (Ao)*	258	31.6	842	5010	6.03
*A. elatius (Ae)*	329	51.9	473	3208	5.34
*D. glomerata (Dg)*	243	52.0	346	2683	4.19
*E. repens (Er)*	297	55.3	476	2775	4.02
*F. rubra (Fr)*	126	30.5	759	10053	5.94
*H. lanatus (Hl)*	326	43.4	503	4332	3.84
*L. perenne (Lp)*	229	46.1	439	4879	5.01
*Clerpin (Cp)*	211	55.0	622	6186	7.23
*Ph. Pratense (Php)*	321	32.2	359	5028	2.32
*P. pratensis (Pp)*	206	34.0	800	6245	6.92
*T. flavescens (Tf)*	316	38.8	739	3841	5.92

In the 4*D* trait space, *trait*_*max*_ is the single trait combination in each species maximizing plant performance.

**Table 2:** Trades-offs between trait pairs in the 4-D trait space as predicted by Gemini for each species.

**Species**	**TD vs SLA**	**TD vs LLS**	**TD vs H**	**SLA vs H**	**SLA vs LLS**	**H vs LLS**	**Relative Sum**
*Ap*	0.25	0.21	-0.05	0.22	-0.68	4.86	0.86
	(r^2^=0.98;-290)	(r^2^=0.90;-310)	(r^2^=0.95;164)	(r^2^=0.88;-2.32)	(r^2^=0.77;408)	(r^2^=0.87;-34)	
*Ao*	0.12	0.23	-0.01	0.1	-1.6	-2.09	0.57
	(r^2^=0.98;-326)	(r^2^=0.60;-742)	(r^2^=0.95;93)	(r^2^=0.98;-6.6)	(r^2^=0.60;743)	(r^2^=0.13;296)	
*Ae*	0.18	0.08	-0.04	0.23	-0.39	1.29	0.52
	(r^2^=0.94;-229)	(r^2^=0.81;-86)	(r^2^=0.96;148)	(r^2^=0.95;-29)	(r^2^=0.75;292)	(r^2^=0.60;113)	
*Dg*	0.13	0.08	-0.05	0.29	-0.64	0.61	0.54
	(r^2^=0.98;-168)	(r^2^=0.91;-131)	(r^2^=0.92;188)	(r^2^=0.86;-1.1)	(r^2^=0.96;239)	(r^2^=0.17;43)	
*Er*	0.13	0.17	-0.04	0.27	-0.94	-2.8	0.67
	(r^2^=0.98;-93)	(r^2^=0.92;-351)	(r^2^=0.98;160)	(r^2^=0.99;-19)	(r^2^=0.96;403)	(r^2^=0.86;-4)	
*Fr*	0.03	0	-0.01	0.19	-1.11	5.2	0.41
	(r^2^=0.90;-99)	(r^2^=0.06;238)	(r^2^=0.85;81)	(r^2^=0.95;5.8)	(r^2^=0.76;444)	(r^2^=0.67;152)	
*Hl*	0.06	0.09	-0.02	0.13	-0.95	4.09	0.47
	(r^2^=0.81;-78)	(r^2^=0.83;249)	(r^2^=0.92;115)	(r^2^=0.98;-12)	(r^2^=0.90;475)	(r^2^=0.91;49)	
*Lp*	0.05	0.04	-0.02	0.33	-0.86	2.2	0.43
	(r^2^=0.97;-29)	(r^2^=0.96;-50)	(r^2^=0.98;134)	(r^2^=0.99;-25)	(r^2^=0.97;334)	(r^2^=0.98;35)	
*Cp*	0.08	0.1	-0.02	0.18	-0.81	1.65	0.42
	(r^2^=0.85;-220)	(r^2^=0.91;-394)	(r^2^=0.84;111)	(r^2^=0.91;2.7)	(r^2^=0.92;422)	(r^2^=0.83;150)	
*Php*	0.07	0.05	-0.01	0.15	-0.4	2.08	0.30
	(r^2^=0.93;21)	(r^2^=0.77;-72)	(r^2^=0.96;18)	(r^2^=0.93;-9.5)	(r^2^=0.75;265)	(r^2^=0.73;103)	
*Pp*	0.02	0.04	-0.01	0.19	-2.21	-1.59	0.41
	(r^2^=0.65;109)	(r^2^=0.81;-624)	(r^2^=0.85;101)	(r^2^=0.80;-3.5)	(r^2^=0.85;401)	(r^2^=0.77;103)	
*Tf*	0.11	0.11	-0.02	0.06	-0.84	-1.59	0.40
	(r^2^=0.90;-131)	(r^2^=0.85;-195)	(r^2^=0.80;75)	(r^2^=0.95;5.9)	(r^2^=0.85;506)	(r^2^=0.77;335)	

The average slope characterizing the co-variation between two traits which minimizes the decline in plant performance is shown for each species (the coefficient of determination and the intercept of the fitted relationship are given in brackets). For a given trait pair (same units as in Table 1), the higher the absolute value of the slope, the stronger the intensity of the trade-off. The relative sum of absolute trait-pair intensity is given at the end of the table, as a proxy of an average coordination between the four traits required to maintain the plant performance. See Table 1 for abbreviations.

In more detail, if one considers for each species a 4*D* (*i*, *j*, *k*, *l*) trait space and keeps the value of the *k* and *l* traits fixed to their observed value, the values of *i* and *j* traits affecting plant performance in a 2*D* space can be systematically explored. The value of the trait *j* maximizing the local plant performance was calculated for each trait *i* value. A linear regression was then fitted across local optimal *i* and *j* trait combinations, thereby defining ridges between the two traits. In a two dimensional trait space, each local ridge between *i* and *j* was defined by a linear relationship of *slope α*_*i,j*_. For *α*_*i,j*_ strictly positive, a local plant performance optimum was reached whenever *j* increased in direct proportion (*α*_*i,j*_) to *i*. Conversely, for *α*_*i,j*_ strictly negative, the local optimum was found for *j* values declining in proportion to *i* (Figure 1). When *α*_*i,j*_ was not different from zero, the local optimum observed for the trait *i* is independent from trait *j* value. Note that this last case could potentially reflect a variety of patterns in the plant performance surface (e.g. if the species response is non-linear). We observed in all cases linear relationships in the plant performance surface.

Using both trait_*max*_ and slope α information, we were able to test our different hypotheses at both intra- and inter-specific levels

#### Analyses at the intraspecific level

First, to assess the maximization of plant performance in response to trait variation (Hypothesis 1), we analyzed graphically the simulated plant total biomass and physiological and morphological processes (particularly, the C:N ratio of plant substrates as the *in-planta* proxy of the coordination between the different plant processes). Then, for each trait and for each N level, we tested whether *trait*_max_ values matched the observed trait values in the field with reduced major axis (RMA) regression. Secondly, we evaluated if trait plasticity in response to an environmental change maximized plant performance (Hypothesis 2). This was achieved by calculating (in the 4*D*-trait space simulated under high-N conditions) the value of each trait that maximized plant biomass locally when the three remaining traits were forced to values observed in the field under low N conditions. As such, we recorded one value per trait that maximized plant biomass under low N conditions based on the *slope α*_*i,j*_ calculated under high N conditions. We compared these predicted trait values with the ones observed under low N conditions using RMA. Note that this procedure offered an independent way to evaluate the model and validate the linkage between *slopes α*_*i,j*_, i.e. trait coordination, and intraspecific trait variation.

#### Analyses at the interspecific level

Firstly, a principal component analysis (PCA) was performed using *trait*_*max*_ values predicted for each species. For each N level, the component coefficients of the two first axes of this PCA were compared with those of a PCA performed with the measured values of the same traits and of the same grass species (Hypothesis 3). Secondly, we tested whether species exhibiting different peaks in performance within the 4*D*-trait space as well as different optimal C:N ratio of plant substrates, were related to plant functional traits and strategy spectra (Hypothesis 4a). Simple regression analyses were conducted between this *in-planta* driver of plant coordination and the *trait*_*max*_ values. Finally, we tested whether species displayed different degrees of trait coordination (Hypothesis 4b). For each species, the sum of the absolute relative values of the slope *α*_*i,j*_, were calculated as a global index (slope *α*_*sum*_) of the intensity of coordination between the four traits that was required to maintain plant performance. For each species, we performed a regression analysis between the values of *slope α*_*i,j*_ and slope *α*_*sum*_ predicted by Gemini and the corresponding observed species trait values under the high N treatment in the field.

All statistical analyses were performed with the Statgraphics Plus (Manugistics, Rockville, MD, USA) software.

## Results

### Effects of traits variations on plant performance simulated by the Gemini model

**Figure 2:**
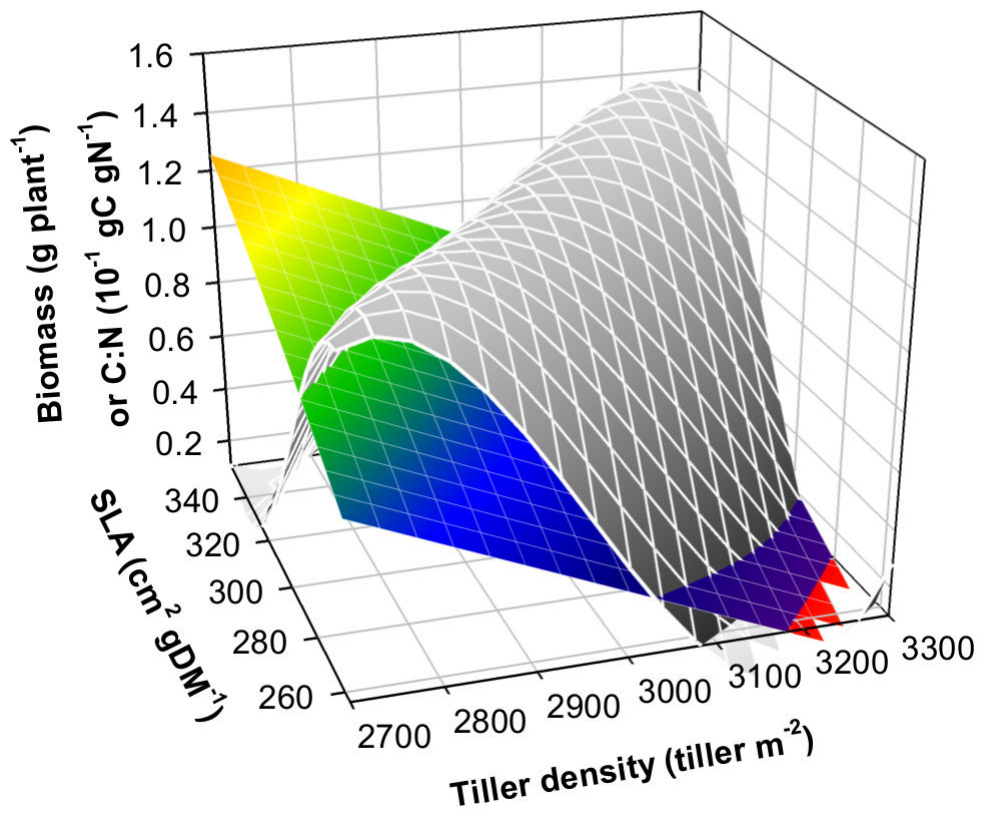
Simulated effects of variations in specific leaf area (SLA) and in tiller density (TD) on annual biomass production (fitted grey mesh plot) and on the C:N ratio of plant substrates (fitted coloured mesh plot) for *Arrhenatherum elatius* in the high N treatment. Values of the two remaining traits (LLS and H) were fixed to the species’ mean trait value observed in the field.

Trait variation had numerous important effects on ecophysiological processes and on plant biomass. The example of *Arrhenaterum elatius* is illustrated in Figures 1 and 2 (see also Figure S1). Within a 2*D* trait space, all binary combinations of the four traits are displayed, thereby showing responses in plant performance to trait variations under the high N treatment (Figure 1). Varying the four traits by up to 30% in absolute value resulted in large changes in plant production (from 0.2 up to 1.3 g DM per plant and per year resulting in 150% of plant performance variation, Figure 1). For each trait combination, a region of high biomass production (displayed in purple in Figure 1) was identified in the 2*D* trait space (see for instance Figure 1A, 1B, 1D). Trait values that locally maximized plant biomass production (or minimized plant performance decline) were shown by regression to follow linear ridges (slope α, see dashed lines in Figure 1). A decline in plant performance outside these ridges indicates negative relationships and potential trade-offs among traits in the 2*D* trait space. For *A. elatius*, we showed that the slope α is specific to each of the six trait-pairs (Table 2), revealing different degree of trait coordination to maintain plant performance.

To help understand underlying mechanisms that determined this trait coordination, we provide a further example, in Figure 2. It shows the effects of variations in tiller density (TD) and in specific leaf area (SLA) on plant performance (i.e. the annual plant production per unit ground area; grey surface) and on the *in-planta* marker of coordination (i.e. the C:N ratio of growth substrates; coloured plane). Along the high biomass ridge (defined by various combinations of SLA and TD), the C:N ratio of plant substrates was maintained in an optimal narrow range (close to 5.3; Table 1). When trait coordination was broken, the simulated plants did not preserve a close to optimal C:N substrate ratio and plant performance decreased. With high and low values of SLA and TD, respectively, plant substrates had a high C:N substrate ratio and plant growth declined due to N substrate limitation and to a C sink limitation caused by reduced morphogenesis. Inversely, with low and high values, respectively, of SLA and TD, the substrate C:N ratio was low and plant growth declined due to a C substrate limitation. Overall, the range of substrate C:N ratio values that maximized plant performance differed across the simulated grass species (Table 1). This result shows that species specific C-N co-limitation was required to attain plant performance.

Within the 4*D* trait space, each species showed a different peak of maximal performance associated with a single combination of *trait*_*max*_ values and C:N substrate ratio (Table 1). Breaking correlations among traits reduced both acquisition and utilization of C and N because of a decline in N-uptake rate and soil exploration; because of a decline in photosynthetic rate and light interception; and, finally, via the changes in the C-N stoichiometry of structural compartments (Figure S1).

### Plant performance, simulated and observed trait variation and co-variation

**Figure 3:**
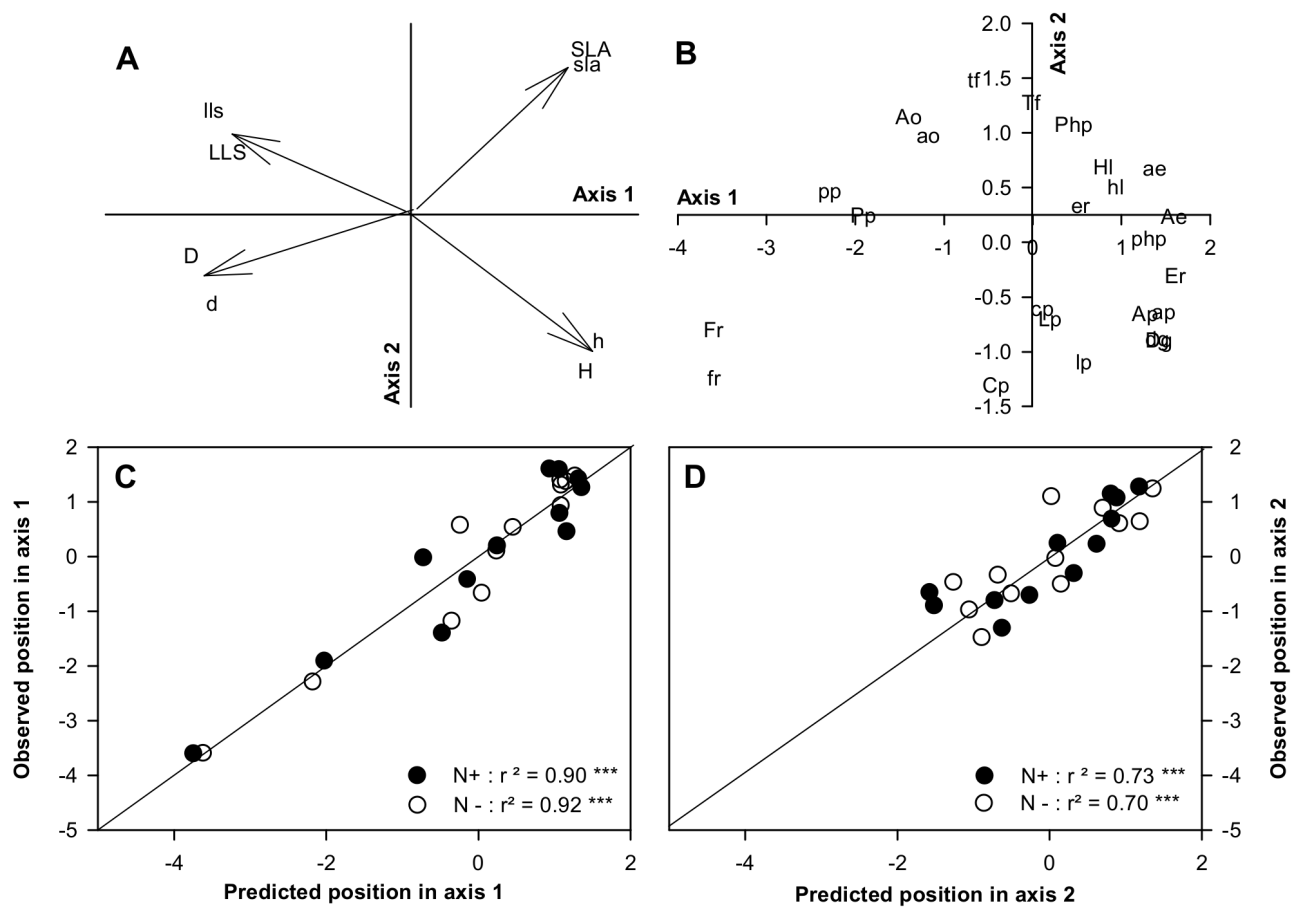
Principal analysis component (PCA) using *trait*_*max*_ values in the low and high N treatments (low cap and high cap, respectively) (A, traits space, B, species space), and relationships between predicted versus observed (i.e. observed species trait values in the field) for axis 1 (C) and axis 2 (D) of the PCA. Abbreviations are: Specific leaf area (SLA); Leaf lifespan (LLS); Plant height (H); Tiller density (TD). See Table 1 for species abbreviation. In all cases, the relative root mean square error (RMSE) is below 10 indicating an accurate agreement between predicted and observed values (***, P < 0.001).

We projected *trait*_*max*_ values from the 4*D*-trait space in a principal component analysis (PCA, Figure 3). The predicted dispersion along the first two axes explained 45% and 32% of total variance, respectively (Figure 3A). This trait manifold represented trait combinations and co-variations which maximized plant performance in the model for all species (Figure 3B). The trait manifold distinguished species with slow leaf turnover and high tiller density (*F. rubra*, *P. pratensis*) from species with high specific leaf area (*Ph. pratense*, *T. flavescens*), on the one hand, and from species with a high stature (*D. glomerata*, *F. arundinacea*), on the other.

To test whether the *trait*_*max*_ values were similar to those observed in the field, we compared predicted versus observed PCA axes coordinates (the latter conducted with observed trait values from the field experiment). For both axes and for the two N supply levels, the regressions were highly significant with slopes not different from one (Axis 1, Figure 3C: y = 0.98 ±0.05 x; y = 1.01 ±0.03 for N+ and N-, respectively; Axis 2, Figure 3D: y = 0.95 ±0.09 x; y = 1.05 ±0.08 for N+ and N-, respectively). That is, trait values maximizing plant growth according to the model (*trait*_*max*_ values) were very close to trait values measured in the field (see also the Figure S2 for a model validation trait by trait, with an overestimation tendency of LLS value prediction).

**Figure 4:**
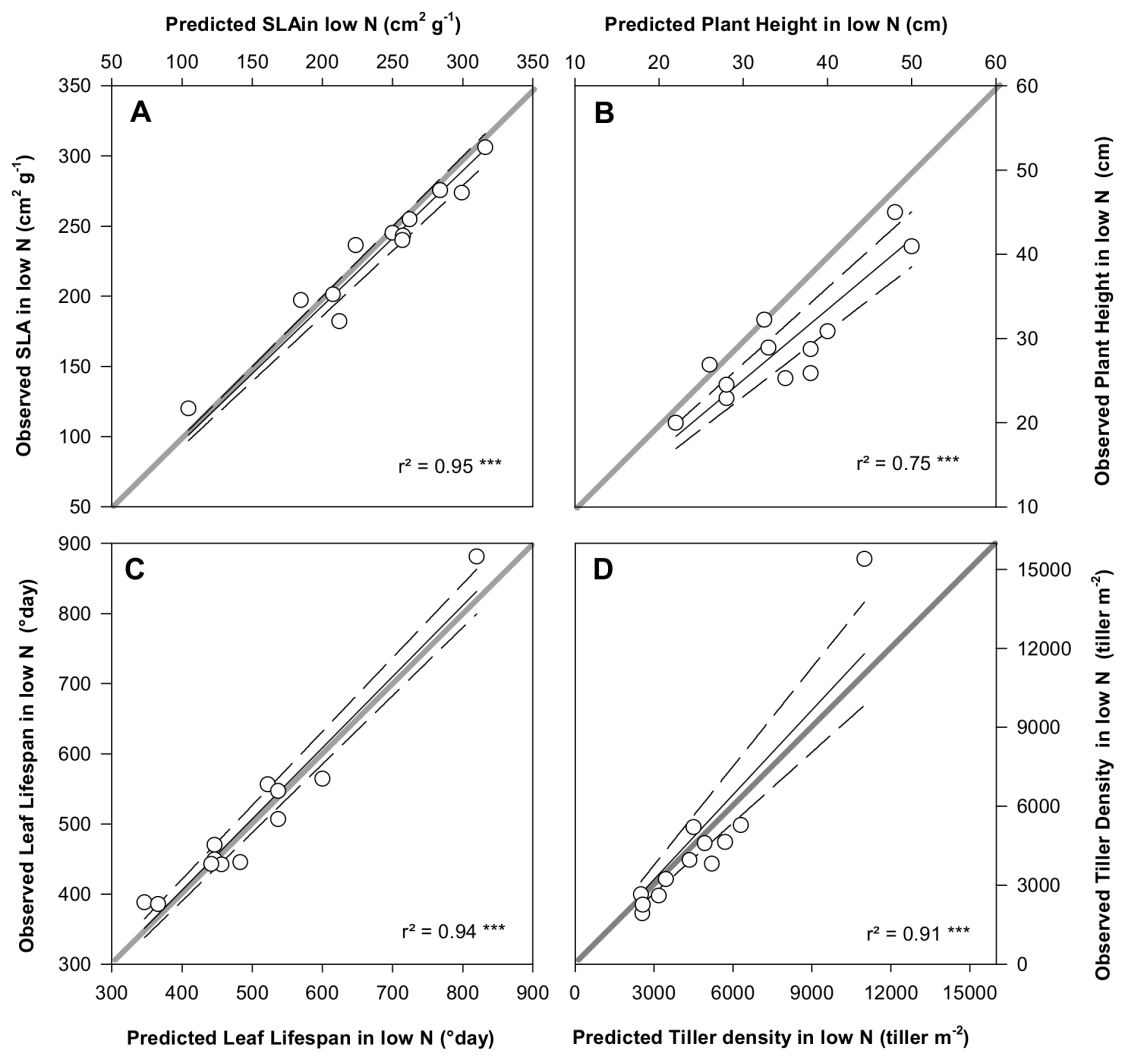
Predicted trait values versus observed trait values in low N conditions for the four traits and for each species, SLA (A), H (B), LLS (C) and TD (D). For a given trait pair, predicted trait values were estimated using the slope αi,j in Figure 1. In all cases RMSE are below 10; ***, P <0.001. See abbreviations in Figure 3. Solid lines are the regressions. Short-dashed lines indicate the confidence interval (at 95%). Grey lines are the 1:1 prediction lines.

To test the optimality of trait plasticity in response to a decline in N availability, we used the intraspecific slopes α, determined under the N+ treatment, to predict the trait values in response to the N- treatment. For the four traits, linear regressions between predicted and observed values were not significantly different from the 1:1 lines (Figure 4, except for the plant Height where the *P*-value = 0.04). Therefore, within species coordinated changes in a suite of traits ensured plant plasticity and plant performance in response to N availability reduction.

### Emergent properties of the 4-D trait space exploration

**Figure 5:**
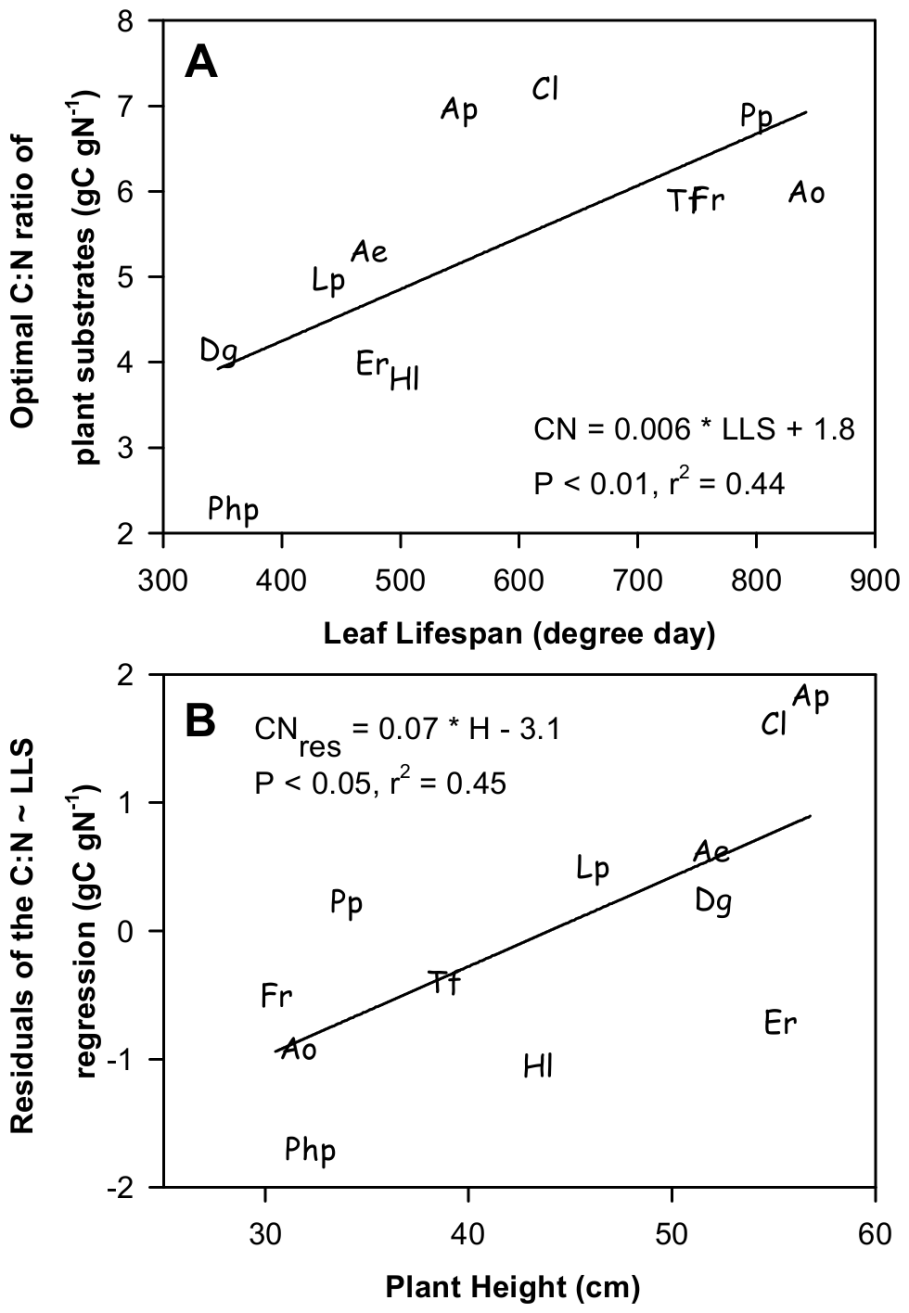
Relationships of the optimal C:N ratio of plant substrates with the trait_max_ values of leaf lifespan and plant height among grass species. A) Linear regression of C:N ratio and LLS; B) Linear regression of the residuals of the C:N vs LLS regression and H. See Table 1 for species abbreviation.

Plant performance tended to be maximized in the Gemini model when the C:N ratio reached an optimal value (Figure 2). Among species, this internal proxy of plant coordination was related to variation in LLS (Figure 5A) and SLA but not in H and TD (data not shown). However, the unexplained variation (i.e. residuals) from this first relationship was significantly and positively related to H (Figure 5B).

A departure from maximum plant performance can be circumvented, or minimized, whenever two traits varied jointly along emergent ridges on the performance response surface (dashed line in Figure 1), reflecting the degree of coordination between traits that preserved plant performance at the intraspecific level. In accordance with trait co-variations observed between species, we observed negative within-species relationships between TD *vs* H and between SLA *vs* LLS - indicating that the trade-offs observed at the interspecific level were conserved at the intraspecific level. However, trait variability within species was also constrained by other trait co-variations, which were not observed at the interspecific level, for instance positive relationships for each of TD vs SLA, TD vs LLS, SLA vs H, and H vs LLS (Table 2).

**Figure 6:**
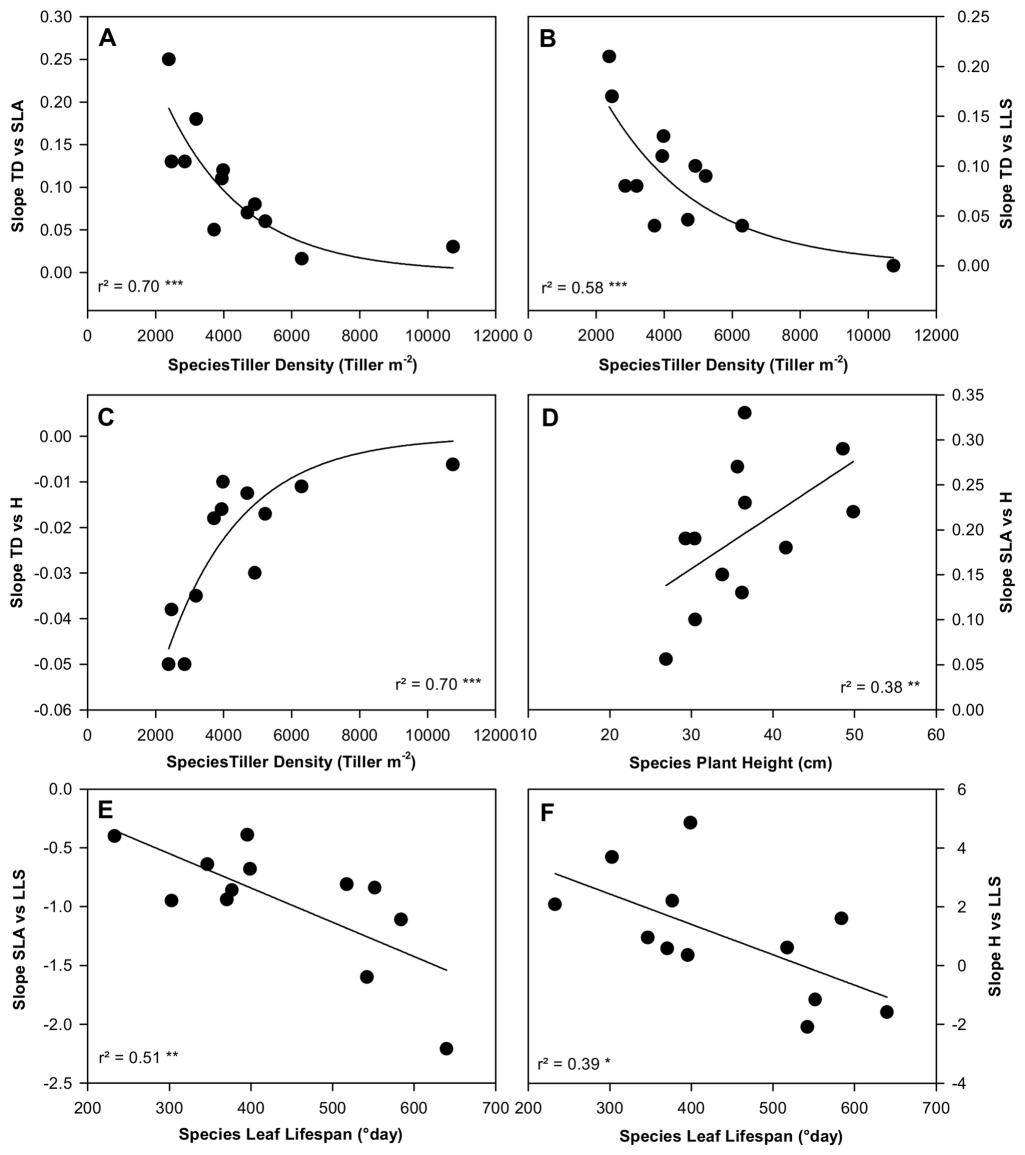
Linkages between observed species trait values and the slope αi,j that each described a set of equally-optimal trait combinations (‘adaptive ridges’) for maximizing plant performance (Figure 1, table 2). Relationships between TD and *slope α*_*i,j*_ for TD *vs* SLA (A), TD *vs* LLS (B), TD *vs* H (C); relationship between H and *slope α*_*i,j*_ for SLA *vs* H (D); relationship between LLS and *slope α*_*i,j*_ for SLA *vs* LLS (E), H *vs* LLS (F). *** P < 0.001, ** P < 0.01, * P < 0.05. See trait abbreviations in Figure 3.

Importantly, we observed large variations in the *slope α*_*i,j*_ (from 5-to 20-fold variation according to the six trait-pairs, Table 2), indicating that trait coordination was species specific. In addition, the slopes *α*_*i,j*_ were related to species *trait*_*max*_ values, indicating that the trait coordination predicted by GEMINI for intraspecific trait variability was related to species trait values measured under high N conditions (species potential trait values). Across species, TD was negatively correlated with the *slope α*_*i,j*_ for TD vs SLA (Figure 6A). Additionally, slopes for TD vs LLS and for TD vs H were correlated negatively and positively, respectively, with TD itself (Figure 6B-C). The slope observed for SLA vs H was positively correlated with H (Figure 6D). Finally, slopes for SLA vs H and for H vs LLS were themselves negatively correlated with LLS (Figure 6E-F). Finally, the sum across the four traits of absolute slope values, is significantly and negatively correlated with TD among species (r^2^ = 0.36, *P* < 0.01, Table 2). Other pair-wise combinations of slopes and trait values were not significantly correlated among species (data not shown).

## Discussion

### Maximization of plant performance: reaching the summit

By using a modeling approach we have explored a 4*D* trait space to investigate the consequences of trait co-variation on plant performance for a variety of grass species. For a given species and under given environmental conditions, the performance surface in response to variation in trait values represented a landscape (*sensu* [44,45]) in which valleys, ridges and summits could be identified (Figure 1, see also 46 for analogy with landscape genetics). In the 4*D* trait space, a single trait combination maximized plant performance (*trait*_*max*_), indicating the occurrence of a single peak in performance per species. For a given species, trait values measured in the field were those which maximized plant performance in the model. Our results are thus in accordance with the optimal trait theory (see 47 for a review), which hypothesizes that plant trait values tend to optimize the capture and utilization of resources.

Under a given environmental condition, different *trait*_*max*_ combinations leading to different biomass optima were found by the Gemini model for the various grass species, which previous work have shown use different functional strategies to acquire and use nitrogen [48]. These different optimal trait combinations (one per species) were not all equally optimal (cf. [29]), but all permit positive plant growth rate in the Gemini model and maximize plant performance in monoculture. In addition, our results show that a given species is able to adapt to lower N availability by adopting a new optimal trait combination (i.e. that maximized plant performance under new environmental conditions). By integrating C and N dynamics from the organ to the whole plant and the complex interactions that act between the size, physiology and morphology of plant parts, the Gemini model was able to reproduce species-specific responses to an environmental change. We were able to better understand the underlying mechanisms of these results through a 4D trait-space exploration.

### Emergent and independent trade-offs at the interspecific level

Interspecific trait covariations predicted by the model were consistent with trade-offs identified in previous empirical studies (e.g. [4,49]). The first trade-off related to plant size (H vs TD) corresponds for tree species to the Corner’s rule ( [50] in [3]), which can be equally observed for grass species [6]. Corner’s rule predicts that species with dense tillering (or dense branching within individuals) have small leaves to avoid overlapping and excess leaf area for light interception (Figure S1A). The second trade-off (SLA *vs* LLS) is a key trade-off underlying the leaf economics spectrum, which runs from ‘conservative’ to ‘acquisitive’ species [2]. Overall, model predictions accord with CSR theory [51], which contrasts tall competitive plants (competitor strategy) from small acquisitive plants (ruderal strategy) from small conservative plants also characterized by dense tillering (stress-tolerator strategy).

The selection of trait values along a given strategy spectrum is sometimes assumed to be established by a combination of environmental filters and competition, and may perhaps also show a phylogenetic signal [3,52,53]. Our results show that in the absence of interspecific competition, the two strategy spectra previously described (Leaf economics spectrum and Corner’s rule) emerged from trait coordination at the intraspecific level. These axes are required to maximize plant performance and minimize allometric and physiological constraints. In addition, assuming evolution selects the value of one given trait, coordination at the intraspecific level forces other traits to move in a concerted fashion. Overall, these results suggest that a trait can be both directly and indirectly selected by evolutionary processes in case it is correlated with another on which selective pressure operates (see 54 for analogy with genetic hitchhiking). Overall, our results suggest that the well-described strategy spectra investigated in the present study might still be under selective pressure and are not only the memory of past selection pressures or phylogenetic affiliation [3,35].

Trait values which maximize species performance have been shown to allow for a within-species homeostasis of the C and N plant substrates, as indicated by a narrow C:N ratio for plant substrates (Figure 2). This result is in line with a recent study showing that the growth of *Festuca paniculata* tussocks tends to be co-limited by both C and N substrates [55]. In addition, different species expressed different optimal C:N ratios that were correlated with between-species trait variation. Interestingly, LLS and SLA (i.e. the leaf economics spectrum) were apparently the primary drivers explaining between-species variation in optimal C:N ratios. This result echoes the theoretical relationship between LLS and dry-mass return [3], that results from the cost-benefit law opposing the respiratory cost of deploying and maintaining dense plant tissue and the benefit to keep plant photosynthetic tissue over long period of time. In addition, at a given LLS, the plant height and, inversely, the tiller density represent a secondary independent control on the maximization of plant performance and on optimal C:N substrate ratios (Figure 5). This reveals that species with high plant stature and low TD may tend to conserve C substrate to sustain high respiratory cost per tiller, in comparison with species that share this substrate among a high number of small interconnected tillers.

Breaking correlations among traits disrupts the acquisition and utilization of C and N (Figure S1) and drives the main *in-planta* marker of coordination (C:N ratio of plant substrates) away from the value that is associated to maximize plant performance (Figure 2). It is sometimes assumed that certain regions of trait space are empty because they would have low performance (e.g. low SLA and low LLS), and other regions are empty because of physiological or genetic constraints (e.g. high SLA and high LLS) [3]. Consistently, our results show that performance may actually be low in regions of trait space that would be expected to have very high performance (e.g. very high SLA and very long LLS) based on leaf economics, but are in fact impossible because of the existence of a second strategy spectrum. For instance, the excess of substrates that could be generated by having both high LLS and SLA syndrome would require their utilization by plant morphogenesis, i.e. either being taller or having more tillers. However, the strong density-dependence relationship involved in the second axis of differentiation imposes an asymmetric negative relationship between H and TD [48], cancelling out the benefits of substrates in excess and decreasing the overall performance. This is captured by the model which simulates the negative density dependence of plant height and tiller number (i.e. self-thinning [33]).

### Plant plasticity follows the ridge and valley of plant performance maximization

A series of co-variations among traits were observed at the intraspecific level. In addition to trade-offs observed at the interspecific level (SLA *vs* LLS, conservation vs exploitation trade-off; H *vs* TD, size, allometric trade-off), trait variation within species was determined by additional trait co-variations (Table 2) that are directly affected by the C dynamics within the plant (Figure 2). For instance, when plant TD or H was increased, the substrate C pool per unit of structural plant mass was reduced. Then, the utilization of C at the individual or population level can be counterbalanced at the leaf level through an increase in SLA value to preserve the overall C balance and an optimal C:N ratio.

The coordination of traits observed at intraspecific level to maintain plant performance was species-specific (Table 2) and determined the direction and the intensity in which each species can be plastic and modify their traits. By using this “map” of trait coordination established under high N conditions, we were able to predict the observed trait variations in response to a decrease of N availability in the field (Figure 4). This important result of our study highlights the fact that species plasticity is not random but follows a species-specific map of trait coordination. For instance, the model predicted that species with high TD, low H, low LLS and high SLA are less penalized by changes in traits away from the optimal values (slope values tending towards zero, Table 2, e.g. *Ph. pratense*) and, thus, display loosely coordinated traits. Plants which tend to be ruderal [56] are predicted to have a larger trait variability than others [57]. Species with the opposite traits syndromes are likely to have a higher C cost in order to adapt their morphology and physiology to environmental change [15]. This result may shed light on the fact that invasive species, which have been shown to be more plastic than native species (Funk, 2008), are often considered as ruderal species [57].

In addition to be species-specific, trait coordination is related to the mean trait values of species at the interspecific level, and therefore is dependent on species functional strategy. For instance, the positive co-variation between TD and SLA is negatively correlated to the tiller density at interspecific level. As such, species with low TD (e.g. *D. glomerata*) requires a high increase of SLA for a given increase in tiller density at intraspecific level. Mechanistically, such species is also characterized by a high plant stature, which asymmetrically increases the C requirement and the light competition for any new individual within the population [16]. At the opposite side of the relationship where species are characterized by high TD (e.g. *F. rubra*), the plant performance seems to be only coordinated by LLS, SLA and H co-variations (slopes α implying TD tended to a zero value, Table 2). In conclusion (see α_sum_ in Table 2), we can contrast the degree of coordination between small stature species for which plant performance maximization and plasticity is mainly coordinated by the leaf economic spectrum, and tall species requiring a higher degree of coordination for traits along both spectra. Such results echo ones observed on tree species, for which the influence of leaf economic spectrum traits on plant performance is most evident in seedlings [58] and decrease systematically with increasing plant size [59].

By identifying trait co-variations observed only at the intraspecific level, our study offers a mechanistic explanation and an explicit test on the origin of trait plasticity often considered as idiosyncratic [14,25]. Similarly with what was observed for interspecific comparison, our study did not explain "why" higher trait variability may give a selective advantage [60,61] but rather provide an explanation on the origins of trade-offs and plant plasticity observed within species in nature. Note that only phenotypic plasticity is considered in the Gemini model, which was sufficient to explain the observed trait variability in our study but this should be completed by the plasticity linked to genotype selection to extend the analysis of plant performance in terms of reproduction.

### A structure-function-diversity model of grassland ecosystems (Gemini)

By using a modeling approach, we have broken the correlations among traits that are usually observed in nature. A genetic approach using, for instance, GMO plants would also be conceivable within a model grass species (e.g. *Brachypodium distachyon* [62]) but would not apply to a large number of plant species. To our knowledge, generating mutant plants with uncorrelated traits has never been done and might be challenging [13]. A modeling approach needs, however, to consider a sufficient degree of realism to investigate the ecophysiological mechanisms that generate trade-offs among traits. Gemini offers the opportunity to test not only the plant responses to trait co-variations but also to investigate the underlying physiological mechanisms at play.

In the model, the maximization of plant performance in response to particular trait combinations is a non-trivial result, arising from multiple but relatively simple equations. The fact that an optimal combination of traits does exist for each species shows: i) from a biological point of view, that species optimize plant performance through different pathways, however based on the same ecophysiological mechanisms and trades-offs; ii) from a modeling point of view, that the complexity of Gemini is efficient for simulating differences of productivity variations among species and across management conditions, as shown by Maire et al. [16]. The capacity of Gemini to predict phenotypic plasticity in response to an environmental change opens new ways to study climate change impacts and disentangle the complex interactions that can occur when multiple climate and soil fertility drivers are manipulated [23,63,64].

We have focused our study on four traits, which have been widely used both in conceptual models and empirical studies, as major functional dimensions of plant species niche [8,26]. We are aware that other strategy spectra may exist to explain the high level of plant species diversity observed in nature. For instance, other spectra may exist for root morphology and N acquisition [48]. Similarly, seed traits such as seed number and size may be linked to another independent spectrum [8]. Future studies are needed to investigate such strategy spectra and understand how they contribute to plant performance.

We have also intentionally chosen to investigate trait relationships in monocultures (intraspecific competition), thereby avoiding the effects of interspecific competition which would have confounded our analysis. However, Gemini has also been shown to simulate adequately the dynamics of plant community structure in three six-species mixtures [33]. As such, it may be able to assemble the four different elements (an optimal strategy and three fitness-limiting factors: resource availability; population density dependence and neighbor frequency dependence) that are required to apply this approach into a game theory perspective [65]. This opens interesting questions on the role of trait coordination on evolutionary stable strategies. Showing different optima of plant performance among species, our results are different from but complementary to the studies that show, under a given environmental condition, equally-optimal trait combination among species, conferring a similar competitive ability and coexistence (e.g. [29]). In a competitive context, not only the competitive ability, through the peak of biomass, but also the niche difference, implying different species peaks, are assumed to drive the community assembly [53]. For instance, on the same grass species pool, Maire et al. [12] have experimentally observed that different trait combinations, defined in non-limiting growth monoculture, are able to predict the success and the coexistence of grass species within different communities and under different management conditions. Altogether, this shows that different plant trait combinations expressed by contrasted species strategies led to different optimal performances that can coexist at the community level (and potentially over long period [66]).

Gemini was parameterized with perennial grass species and further developments are required to extend the results to other grassland plant families (e.g. forbs and legumes) and other environmental conditions (e.g. water). Gemini does not incorporate a plant reproduction stage and this would be required to fully simulate demographic processes (e.g. [67]). For instance, ontogeny has been shown to impact the relationship between trait variability and the optimization of plant performance [68]. By extending our model to integrate explicitly reproductive stages and plant ontogeny, new insights on processes that determine the evolution of ecological specialization could be gained [69]. In this context, the very recent identification of genes implicated in the morphological diversification of plants [13] may help to design a future mechanistic approach, coupling a genetic framework (e.g. based on adaptive dynamics and the identification of genetic constraint [70,71]) with morphological and physiological constraints predicted by our model.

## Conclusion

By using a model that considers physiological and morphological processes, from organs to the canopy level, we were able to propose a mechanistic and causal explanation for the origin of trade-offs among traits observed in nature at both intra- (trait variability) and inter-specific level. At the interspecific level, each species can be viewed as an island which locally maximizes plant performance in a multidimensional trait space. Within species, we identified a series of trade-offs that complement those observed at the interspecific level. These trade-offs determined the ability of a species to adapt its morphology and physiology in response to an environmental change such as N deprivation. We demonstrated that plasticity can be related to species strategies (functional traits syndrome), for instance small and fast growing plants were predicted to be more plastic than others. Overall, observed trait correlations appear to be determined by cost and benefit relationships [72]. Species tend to coordinate leaf, root and whole plant processes leading to a plant resources co-limitation in order to minimize their costs (C and N allocation to structure and function) and maximize their benefits (resource acquisition). As such, our study highlights the importance of C and N co-limitation processes at the leaf and plant levels, which are likely to determine morphological diversification among and within plant species. 

## Supporting Information

Text S1 **Protocols for traits measurements and model parameterization.**
(DOC)

Table S1 **Details on virtual experiment design.** Observed, minimum, maximum and step values used in the virtual experiment. Simulations explored 10 step values per trait and per species between minimum and maximum observed boundaries (+ or -30% around the traits value); in addition to the 10 steps, a simulation with the observed trait value in the field was also performed for each species. Abbreviations: *SLA*, specific leaf area; *H*, maximal plant height; *LLS*_*0*_, minimum leaf lifespan; *TD*_*0*_, initial tiller density.
(DOC)

Figure S1 **Relationship between growth and eco-physiological processes of *Arrhenatherum elatius*.** Example of model output across the 4D trait space: relationship between eco-physiological processes and biomass production for *A. elatius* in the high N level treatment. Each point represents a simulation run for a particular trait combination. The eco-physiological variables are the radiation interception (A), net photosynthesis (B), root N uptake rate (C), specific root area (D), substrate allocation coefficient P between root and shoot structure (E), substrate allocation coefficient Q between shoot structure and leaf photosynthetic proteins (F), nitrogen use efficiency (G) and radiation use efficiency (H). Net photosynthesis, N uptake rate (*Su*) and specific root area (SRA) were normalized between 0 and 1, one being the maximal value in the data set. Regression statistics between biomass and each eco-physiological process (r^2^ and p value: ***, P < 0.001) are provided. A variance decomposition analysis allowed ranking variable pairs for their relative weights (%var) for plant biomass production. We compared: light interception (%var = 9) vs. net photosynthesis (%var = 91); *Su* (%var = 16) vs. SRA (%var = 84); P (%var = 3) vs. Q (%var = 97); and NUE (%var = 1) vs. RUE (%var = 99).
(TIF)

Figure S2 **Relationship between predicted and observed trait values for SLA (A), Plant Height (B), Leaf Lifespan (C) and Tiller density (D) in low and high N treatments.** In all cases relative root mean square error (RMSE) is below 10 indicating an accurate agreement between predicted and observed values; *** P < 0.001; ** P < 0.01.
(TIF)
