# Supplementary material for: Disentangling Coordination among Functional Traits Using an Individual-Centred Model: Impact on Plant Performance at Intra- and Inter-Specific Levels
Source: PLoS One. 2013 Oct 9;8(10):e77372. doi: 10.1371/journal.pone.0077372 (PMC3793938; doi:10.1371/journal.pone.0077372)
Supplement: Text S1 — Protocols for traits measurements and model parameterization. (DOC) [file pone.0077372.s001.doc]

**Text S1 Protocols for traits measurements and model parameterization**

**Site and species collection**

The experiment was established in an upland area of central France (Theix, 45º43’N, 03º01’E, 870 m a.s.l.) on granitic brown soil (Cambic soil, FAO, 43% sand, 36% silt, 21% clay, pH(H_2_O) 6.2, 5.2% OM). The local climate is semi-continental with a mean annual temperature of 9ºC ranging from 1°C in January to 20°C in August, and an average annual precipitation of 760mm.

We studied 12 C_3_ grasses (*Alopecurus pratensis*, *Anthoxanthum odoratum*, *Arrhenatherum elatius*, *Dactylis glomerata*, *Elytrigia repens*, *Festuca arundinacea*, *Festuca rubra*, *Holcus lanatus*, *Lolium perenne*, *Phleum pratense*, *Poa pratensis* and *Trisetum flavescens*), co-occurring in semi-natural mesic grasslands of the French Massif Central region (Louault *et al.*, 2005). These species account for more than 80% of the total biomass of these meadows. We set up a factorial three-block design in spring 2002 that comprised 72 monocultures of the 12 grass species. Experimental plots of 2.8x1.5m were sown homogenously in eight rows (18cm apart). The six-species mixtures were sown with the same seed density (100 seeds species^-1^ m^-2^) as the monocultures (600 seeds m^-2^). Shortly after the beginning of the experiment (end of year 2002), vegetation cover was well established in all plots with tiller density similar to semi-natural grasslands (between 2000 and 5000 tillers m^-2^).

Two management conditions were established with two levels of mineral N supply (NH_4_NO_3_, 12 and 36 gN m^-2^ yr^-1^, N- and N^+^ treatments, respectively) in monocultures. The monocultures were managed to simulate conditions found in productive grassland (common hay meadows). From spring 2002 onwards, all plots were cut three times per year to a height of 6 cm. Phosphorus and potassium were supplied in spring at non-limiting rates for growth. When soil water content was below 10%, all plots were irrigated (see Pontes *et al.* 2007 for full details).

**Dry matter yield**

At each cutting date, the fresh harvested biomass of each individual plot was automatically collected by the mower and weighed. A subsample was immediately taken, weighed and dried at 60 8C for 48 h to determine the dry matter (DM) content of the harvested biomass and calculate the ANPP of each plot (g DM m^-2^). The annual ANPP (g DM m^-2^ year^-1^) was calculated as the sum of the three cuts taken each year.

**Plant functional traits measurement**

Leaf traits were measured in June and in September 2003 and 2004 and in September 2006, 3 weeks after a cut in two management conditions, according to a standard protocol (Cornelissen et al. 2003). Ten tillers were collected at random in each plot, avoiding 20 cm edges, cut with scalpels at ground level and kept in a cold box. In the laboratory, at plant level, the vegetative plant height was first measured (H). The tiller base was cut in de-ionized water and was then placed at 4 °C in the dark for at least 6 h to allow for full rehydration (Garnier *et al* 2001). After rehydration, the lamina of the youngest fully expanded leaf of each of the ten individuals was weighed and their area was measured with an electronic planimeter (LI 3100, Li-cor, Lincoln, NE, USA). The leaves were then oven dried at 60 °C for 48 h and weighed. Specific leaf area (SLA) was calculated as the ratio of lamina area and lamina dry mass.

Leaf lifespan (LLS, d) was calculated as the product of the leaf appearance interval (or phyllochron, PH, °C d mature leaf^-1^*)* and of mature leaf number (NM, leaf) (Lemaire & Agnusdei 2000), as NM is constant during the growing season for our species (Marriott, Barthram & Bolton 1999). This product was then multiplied by the mean air temperature of the sampling period (17.7 °C). PH and NM were determined, in each plot, in summer on eight labelled tillers following Carrere *et al.* (1997). PH was calculated as the thermal time-span in degree-days (accumulated temperature above 0°C leaf^-1^) between the appearances of two successive newly emerged leaves (Lemaire & Agnusdei 1999).

The tiller density (TD) per unit ground area was determined every 2 months, at each cutting date, from the mean tiller mass and the harvested dry matter. In each plot, 16 tillers were sampled at random, cut at a height of 6 cm, dried at 60 8C for 48 h and weighed. The total tiller density (TD) was calculated as the ratio of the harvested DM to the mean individual tiller mass.

**Model parameterization**

A detailed list of all 132 equations, as well as the 187 variables and the 100 default parameters is available at www1.clermont.inra.fr/urep/modeles/gemini.htm. The four studied traits refer specifically to the morphogenesis module. They all have an indirect impact on C and N internal fluxes within the plant through the coordination between the physiology and the morphogenesis Gemini modules. A brief review of their implication in model equations is given hereafter.

Variation in specific leaf area (SLA) was achieved by changing leaf dry-matter content (LDMC). For convenience, the equation presented hereafter was modified to be expressed as a function of SLA, compared with the original equation firstly published in Soussana et al. 2012. Then, SLA is implied in the transformation of the leaf elongation by morphogenesis module into assimilates for the physiological module of Gemini and vice versa. The assimilate demand (C and N substrates) is based on the increment in growing leaf volume (Eqn 1), which is calculated from leaf extension rate (*dL*) and from current leaf length (*L*) according to the allometry between leaf length and leaf area, and leaf volume (*a*_AL_, *b*_Wl_, respectively) and a constant fraction of sheath (*F*_sheath_).

$DV_{\text{pot}}=\sum_{\text{leaf=Grow}} \left[\frac{a_{\text{AL}}\cdot\left(1+F_{\text{sheath}}\right)}{SLA}\cdot\left(\left(L+dL_{\text{pot}}\right)^{b_{\text{WL}}}-L^{b_{\text{WL}}}\right)\right]$ Eqn 1

The leaf lifespan (LLS) is implied in the leaf emission, elongation and senescence rates. A leaf appears when two conditions are met: i) there are no leaves growing; ii) one phyllochron (*ph,* the leaf appearance interval which is proportionally linked to LLS) has elapsed since the last leaf emission event. The leaf senescence is based on leaf area (*area*, Eqn 2). It varies exponentially with air temperature (*T*_a_ is the air temperature, *T*_s_ is the base temperature at which growth starts and b_LER_, the exponential coefficient) and also inversely to LLS and the number of growing leaves (*ng*).

$area_{\text{Sen}}=\frac{area\cdot T_{\text{S}}\cdot\left(T_{\text{a}}/T_{\text{S}}\right)^{b_{\text{LER}}}}{LLS\cdot ng\cdot T_{\text{a}}}$ Eqn 2

The plant height (H) variation is achieved through the variation of the maximal length (*L*_0_) that a leaf can achieve. The maximal leaf length is implied in the potential leaf elongation rate depending on the air temperature as follows.

$dL_{\text{pot}}=L_{0}\cdot T_{\text{S}}\cdot\left(T_{\text{a}}/T_{\text{S}}\right)^{b_{\text{LER}}}/\left(ng_{\text{leaf}}\cdot LLS\cdot T_{\text{a}}\right)$ Eqn 3

The plant height is then calculated according to the shoot architecture module. For grass species, a concave shape of a leaf lamina is modelled by a rectangular hyperbole (Eqn 4a,4b), which is parameterised according to leaf length (*L*) and to canopy height (H). As a result of a system of two equations with two unknowns solved by an optimisation procedure, when the canopy height increases the grass leaves become more and more erect, which have consequences for the radiation balance.

$H(L)=\frac{V_{\text{m}}\cdot L}{L+K_{\text{m}}}$ Eqn 4a $V_{\text{m}}=1.66\cdot H^{0.9}$ Eqn 4b

Where *V*_m_ is the dependence of grass leaf shape on the canopy height and *K*_m_ is the dependence of grass leaf shape on the canopy density and on C.

Finally, the tiller density (D), which is a state variable in the Gemini model, is implied in the species population dynamic. Tiller density directly impacts the calculation of the axis appearance rate. This rate is either limited by the plant morphogenesis (assimilate demand, Eqn 5) or by the plant physiology (assimilate supply, Eqn 6). Considering no assimilate limitation, the axis appearance rate is the product of the site filling rate (*sfr*, the potential maximum site filing rate; *ltgl*, the proportion of radiation) and the leaf appearance rate (the ratio between the air temperature and the phyllocron).

$dtf=D\cdot ltgl\cdot sfr\cdot\frac{T_{\text{a}}}{ph}$ Eqn 5

When substrate (C or N) limitation occurs, a substrate cost for which an axis reaches the autotrophy is calculated for the population (*C*til_demand and *N*til_demand) and compared with the availability of C and N substrates (*W*_C_ and *W*_N_, respectively). A clonal integration parameter (*intcl*) modulates this effect.

$ntf_{\text{C}}=D\cdot intcl\cdot\frac{T_{\text{a}}}{ph}\cdot\frac{W_{\text{C}}}{C_{\text{til\_demand}}}$ Eqn 6a $ntf_{\text{N}}=D\cdot intcl\cdot\frac{T_{\text{a}}}{ph}\cdot\frac{W_{\text{N}}}{N_{\text{til\_demand}}}$ Eqn 6b

Where ntf_C_ and ntf_N_ are, then, the appearance axis rates limited by the supply of C and N substrates, respectively.

**References**

Carrère P, Louault F, Soussana JF (1997) Tissue turnover within grass-clover mixed swards grazed by sheep. Methodology for calculating growth, senescence and intake fluxes. Journal of Applied Ecology 34: 333-348.

Cornelissen JHC, Lavorel S, Garnier E, Diaz S, Buchmann N et al. (2003) A handbook of protocols for standardised and easy measurement of plant functional traits worldwide. Aust J Bot 51(4): 335-380.

Garnier E, Laurent G, Bellmann A, Debain S, Berthelier P et al. (2001) Consistency of species ranking based on functional leaf traits. New Phytol 152(1): 69-83.

Lemaire G, Agnusdei M (2000) Leaf tissue turnover and efficiency of herbage utilization. Grassland ecophysiology and grazing ecology. Wallingford UK: CABI Publishing. pp. 265-287.

Louault F, Pillar VD, Aufrere J, Garnier E, Soussana JF (2005) Plant traits and functional types in response to reduced disturbance in a semi-natural grassland. J Veg Sci 16(2): 151-160.

Marriott CA, Barthram GT, Bolton GR (1999) Seasonal dynamics of leaf extension and losses to senescence and herbivory in extensively managed sown ryegrass-white clover swards. J Agric Sci Cambridge 132: 77-89.

Pontes LS, Carrere P, Andueza D, Louault F, Soussana JF (2007) Seasonal productivity and nutritive value of temperate grasses found in semi-natural pastures in Europe: responses to cutting frequency and N supply. Grass Forage Sci 62(4): 485-496.
