# Supplementary material for: Disentangling Coordination among Functional Traits Using an Individual-Centred Model: Impact on Plant Performance at Intra- and Inter-Specific Levels
Source: PLoS One. 2013 Oct 9;8(10):e77372. doi: 10.1371/journal.pone.0077372 (PMC3793938; doi:10.1371/journal.pone.0077372)
Supplement: Table S1 — Details on virtual experiment design. Observed, minimum, maximum and step values used in the virtual experiment. Simulations explored 10 step values per trait and per species between minimum and maximum observed boundaries (+ or -30% around the traits value); in addition to the 10 steps, a simulation with the observed trait value in the field was also performed for each species. Abbreviations: SLA, specific leaf area; H, maximal plant height; LLS 0, minimum leaf lifespan; TD 0, initial tiller density. (DOC) [file pone.0077372.s002.doc]

**Table S1** **Details on virtual experiment design**: Observed, minimum, maximum and step values used in the virtual experiment. Simulations explored 10 step values per trait and per species between minimum and maximum observed boundaries (+ or – 30 % around the traits value); in addition to the 10 steps, a simulation with the observed trait value in the field was also performed for each species. Abbreviations: *SLA*, specific leaf area; *H*, Plant Height; *LLS*, leaf lifespan; *D_0_*, initial tiller density.

| **Species** | **Values** | **SLA** | **LLS** | **H** | **TD_0_** |
| --- | --- | --- | --- | --- | --- |
| *Alopecurus pratensis* | Obs. | 247.0 | 398.94 | 56.98 | 2400 |
|  | Min. | 355.1 | 402.6 | 40.00 | 2200 |
|  | Max. | 239.8 | 622.2 | 58.00 | 2600 |
|  | Step | 11.5 | 21.96 | 1.80 | 40 |
| *Anthoxanthum odoratum* | Obs. | 267.4 | 542.168 | 31.55 | 4250 |
|  | Min. | 346.3 | 475.8 | 20.00 | 4450 |
|  | Max. | 246.4 | 951.6 | 32.00 | 5050 |
|  | Step | 10.0 | 47.58 | 1.20 | 60 |
| *Arrhenatherum elatius* | Obs. | 316.8 | 395.524 | 43.09 | 3188 |
|  | Min. | 356.7 | 292.8 | 34.00 | 2700 |
|  | Max. | 249.9 | 475.8 | 66.00 | 3300 |
|  | Step | 10.7 | 18.3 | 3.20 | 60 |
| *Dactylis glomerata* | Obs. | 240.7 | 346.48 | 52.02 | 2855 |
|  | Min. | 253.5 | 183 | 48.00 | 2500 |
|  | Max. | 168.1 | 366 | 68.00 | 2900 |
|  | Step | 8.5 | 18.3 | 2.00 | 40 |
| *Elytrigia repens* | Obs. | 279.2 | 370.392 | 48.85 | 2448 |
|  | Min. | 317.2 | 183 | 46.00 | 2500 |
|  | Max. | 234.4 | 475.8 | 66.00 | 2900 |
|  | Step | 8.3 | 29.28 | 2.00 | 40 |
| *Festuca rubra* | Obs. | 119.5 | 583.892 | 29.27 | 10700 |
|  | Min. | 181.5 | 512.4 | 16.00 | 8000 |
|  | Max. | 119.5 | 805.2 | 36.00 | 10000 |
|  | Step | 6.2 | 29.28 | 2.00 | 200 |
| *Holcus lanatus* | Obs. | 330.3 | 302.804 | 48.38 | 4242 |
|  | Min. | 427.4 | 219.6 | 30.00 | 3300 |
|  | Max. | 296.6 | 512.4 | 54.00 | 4500 |
|  | Step | 13.1 | 29.28 | 2.40 | 120 |
| *Lolium perenne* | Obs. | 231.9 | 376.736 | 40.88 | 4671 |
|  | Min. | 239.8 | 292.8 | 38.00 | 4300 |
|  | Max. | 176.2 | 439.2 | 58.00 | 5000 |
|  | Step | 6.4 | 14.64 | 2.00 | 70 |
| *Lolper var. Clerpin* | Obs. | 254.8 | 517.28 | 41.60 | 5850 |
|  | Min. | 287.4 | 329.4 | 30.00 | 5700 |
|  | Max. | 196.7 | 622.2 | 66.00 | 6300 |
|  | Step | 9.1 | 29.28 | 3.60 | 60 |
| *Phleum pratense* | Obs. | 307.2 | 232.776 | 33.80 | 4700 |
|  | Min. | 363.1 | 183 | 28.00 | 3600 |
|  | Max. | 266.9 | 366 | 46.00 | 5200 |
|  | Step | 9.6 | 18.3 | 1.80 | 160 |
| *Poa pratensis* | Obs. | 211.6 | 790.072 | 35.25 | 6300 |
|  | Min. | 233.4 | 512.4 | 16.00 | 5200 |
|  | Max. | 197.0 | 951.6 | 40.00 | 6300 |
|  | Step | 3.6 | 43.92 | 2.40 | 110 |
| *Trisetum flavesscens* | Obs. | 274.2 | 551.684 | 32.23 | 4000 |
|  | Min. | 385.0 | 439.2 | 20.00 | 3000 |
|  | Max. | 226.0 | 768.6 | 40.00 | 4500 |
|  | Step | 15.9 | 32.94 | 2.00 | 150 |
