# Supplementary figures and images for: Disentangling Coordination among Functional Traits Using an Individual-Centred Model: Impact on Plant Performance at Intra- and Inter-Specific Levels
Source: PLoS One. 2013 Oct 9;8(10):e77372. doi: 10.1371/journal.pone.0077372 (PMC3793938; doi:10.1371/journal.pone.0077372)

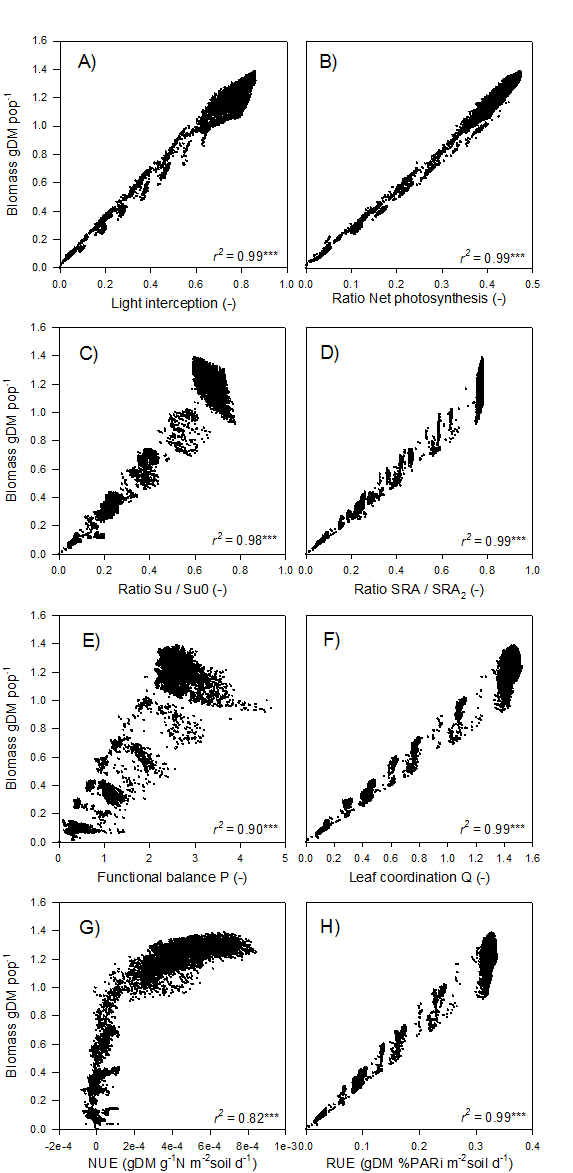

Supplement: Figure S1 — Relationship between growth and eco-physiological processes of Arrhenatherum elatius. Example of model output across the 4D trait space: relationship between eco-physiological processes and biomass production for A. elatius in the high N level treatment. Each point represents a simulation run for a particular trait combination. The eco-physiological variables are the radiation interception (A), net photosynthesis (B), root N uptake rate (C), specific root area (D), substrate allocation coefficient P between root and shoot structure (E), substrate allocation coefficient Q between shoot structure and leaf photosynthetic proteins (F), nitrogen use efficiency (G) and radiation use efficiency (H). Net photosynthesis, N uptake rate (Su) and specific root area (SRA) were normalized between 0 and 1, one being the maximal value in the data set. Regression statistics between biomass and each eco-physiological process (r2 and p value: ***, P < 0.001) are provided. A variance decomposition analysis allowed ranking variable pairs for their relative weights (%var) for plant biomass production. We compared: light interception (%var = 9) vs. net photosynthesis (%var = 91); Su (%var = 16) vs. SRA (%var = 84); P (%var = 3) vs. Q (%var = 97); and NUE (%var = 1) vs. RUE (%var = 99). (TIF) [file pone.0077372.s003.tif]
